# 

**Published:** 1818-10-01

**Authors:** 


### To STUDENTS.

Dr. James Johnson, Editor of the Medico-Chirurgical Journal, &c. will admit into his House, No. 1, Albany, Piccadilly, Two Pupils, (one of whom is engaged) for the purpose of superintending their Studies, and initiating them in British and Foreign Medical and Surgical Literature. Any Gentleman designed for a Tropical field of Practice, will find this an eligible Situation, while completing his Studies. The Terms are moderate, and advantageous to the Pupil.

### CORRESPONDENCE.

Dr. Moulson's Cases of Loss of Power in the Voluntary Muscles, in our next.

Dr. Cookworthy's very interesting Case of Extra Uterine Conception; Dr. Dickson's Case of Urticaria; and Dr. Porter's valuable paper on Hydrocephalus with Cases and Dissections, are received, and will certainly appear in the ensuing Number; as will the able paper of Mr. Niel; Rostan's Cases of active Aneurism of the Heart, various other Favours, and several most important Reviews.

The Review of Dr. Burrows's Pamphlet was accidentally mislaid, while the Editor was removing from the Country to London, and the Circumstance was not noticed in time for the present Number.

The very interesting Case, with Appearances on Dissection, of internal Visceral Disease, by Mr. Jos. Stilon, of Malta, is received; and will appear, if possible, in our next Number.

To Dr. Johnson of Weymouth, and Mr. Fairchild of Rocheford, the Editor's grateful acknowledgements are due; as also to Mr. Norman of Langport, and Mr. Sankey of Dover.

Mr. Colville's Case of Spontaneous Cure of Ascites is received.

Dr. Carbutt's Hints, and also those of Dr. Lyon, are under consideration; but it is extremely difficult, and sometimes dangerous, to make alterations that appear quite easy in principle.

We have made inquiries, and can inform L. that B. is not dead. "Amicus" will see some of his suggestions *anticipated* in this Number; but other of his wishes are unreasonable, and his scruples fastidious to a degree. His complaints would be pronounced by any Literary Court Martial in Europe—"frivolous and vexatious."

It is hoped that it will not appear ungenerous, to request that Letters and Communications relative to the Journal may be transmitted free of expense. This is a trifling tax on the individual; but the omission of it, *in the aggregate*, becomes a serious affair to the Editor. He gratefully acknowledges, however, the kind consideration of *most* of his Correspondents in this particular. Books for Review are requested to be sent early.

The Editor proposes to publish the Names, Residences, and official Appointments of all Subscribers to this Journal *quarterly*, as they arrive, and *annually*, in a complete alphabetical List, with each volume.

---

The first Series of the Medico-Chirurgical Journal, in five volumes, conducted by Drs. Shearman, Johnson, and Palmer, and comprehending a complete Epitome of the Medical Literature of the period which it embraces, may be had of Mr. W. Thorne, Printer, Red Lion Court, Fleet Street; of the Publishers, Burgess and Hill, or any of the Medical Booksellers, price 3l. 15s. boards.

*Additional Subscribers to the Quarterly Form of the Medico-Chirurgical Journal.*

(Since last Quarter.)

Alcock, Mr. Piccadilly
Barton, Mr. Manchester
Bampfield, Mr. 31st Reg.
Berryman, Mr. Penzance
Bowman, Mr. D. Harley Street
Brigham, Mr. Manchester
Burgess, Mr. Windmill Street
Carbutt, Dr. Manchester
Carver Mr. 18th Reg.
Clifton, Mr. London
Curtis, J. H. Esq. Soho Square
Davidson, Mr Alexander
Davis, Dr. George Street, Hanover Square
Ellerby, Mr. Cannon Street
Fairchild. Mr. Rocheford, Essex
Fallofield, Mr. Albemarle Street
Fiddes, Mr. 85th Regiment
Two Assistants, ditto
Forbes, Dr. Penzance
Gardener, Rev. F. Neston, Cheshire
Gray, Dr. R. N.
Grattan, Dr. Censor of the Royal College of Physicians, Dublin
Gunning, Mr. John, F.L.S. Surgeon-Extraordinary to the King, and Surgeon to Saint George's Hospital, Lower Grosvenor Street
Harper, Mr. W. Manchester
Hart, Dr. Red Lion Square
Hendrickson, Mr.
Howel, Dr. Neath, Glamorganshire
Howship, Mr. Mill Street, Hanover Square
Johnson, Edward, M. D. Physician to His Royal Highness the Duke of Cambridge, and Physician to the Dispensary at Weymouth
Jordan, Mr. J. Manchester
Lillies, Mr. George, R. N.
Luke, Dr. Argyle Street
Lyon, Dr. Manchester
M'Grigor, Sir James, M.D. Director General of the Army Medical Department
Medical Committee of the Manchester Infirmary
Medico-Chirurgical Society, London
Mortimer, Mr. Torrington, Devon
Nicol, Dr. Staff-Surgeon, Sierra Leone
Norman, Mr. Langport
North, Mr. Seymour Place
Palmer, Dr. Tamworth
Punshon, Mr. Staff-Surgeon, Ceylon
Roberts, Mr. Manchester
Roscoe, Mr. Richard
Royle, Mr. Sloane Street
Sankey, Mr. Dover
Stilon, Mr. Joseph, H. M. Naval Dock Yard, Malta
Walker, Dr.
Yates, Dr. Thomas

---

☞ It is to be particularly observed, that the Subscribers to this Journal are to procure and settle for it through the medium of their own Booksellers, in the same way as any other periodical publication is ordered from London by the provincial Bookseller.

JAMES JOHNSON.

*Albany, Piccadilly, Sept.* 29, 1818.

